# High Performance Rotating Triboelectric Nanogenerator with Coaxial Rolling Charge Pump Strategy

**DOI:** 10.3390/mi14122160

**Published:** 2023-11-26

**Authors:** Congcong Hao, Bowen Qi, Zekun Wang, Mingzhe Cai, Juan Cui, Yongqiu Zheng

**Affiliations:** 1 Key Laboratory of Instrumentation Science & Dynamic Measurement Ministry of Education, North University of China, Taiyuan 030051, China; qbw769844210@163.com (B.Q.); wangzekun0503@163.com (Z.W.); 19834044879@163.com (M.C.); cuijuan@nuc.edu.cn (J.C.); 2 State Key Laboratory of Electronic Thin Films and Integrated Devices, University of Electronic Science and Technology of China, Chengdu 611731, China

**Keywords:** triboelectric nanogenerator, charge pump, coaxial rotation, wind energy

## Abstract

With the development of society and the advancement of technology, the emergence of the Internet of Things (IoT) has changed people’s lifestyles and raised the demand for energy to a new level. However, there are some drawbacks in terms of energy supply for IoT sensors, such as limited battery capacity and limitations in replacement and maintenance. Therefore, it has become urgent to develop a sustainable green energy source (wind energy) using the surrounding environment. Meanwhile, triboelectric nanogenerators (TENGs) with advantages such as flexible structure, low manufacturing cost, and environmental friendliness provide enormous potential for constructing self-powered sensing systems. In this work, we present a novel coaxial rolling charge pump TENG (CR-TENG) based on wind energy to enhance the output performance and durability. The rolling friction charge pump TENG directly injects positive and negative charges into the main TENG, which is more wear-resistant compared to sliding friction, and greatly increases the charge density and output power. In addition, the charge pumping part and the main TENG adopt the coaxial design, reducing the complexity of the structural design. On comparing the output performance of the CR-TENG under the initial state, rectifier bridge supplemental charge strategy, and charge pump supplemental charge strategy, results shown that the output voltage performance of the CR-TENG can be improved by 5800% under the charge pump supplemental charge strategy. Moreover, the output performance of the CR-TENG remains stable after 72,000 cycles. The output power of the CR-TENG can reach 1.21 mW with a load resistance of 3 × 10^7^ Ω. And the CR-TENG can charge a 0.1 μF capacitor to 5 V in just 1.6 s. This work provides new insights for the rotary durable high output charge pump compensating a triboelectric nanogenerator and demonstrates the important potential of harvesting environmental energy to supply intelligent IoT nodes.

## 1. Introduction

The emergence of the Internet of Things (IoT) has changed people’s lifestyles and raised energy demand to a new level [1,2,3,4]. IoT sensor nodes are usually powered by batteries [5,6,7]. Battery power has some inherent drawbacks, such as limited battery capacity and constraints in replacement and maintenance [8,9]. Therefore, developing sustainable and green energy sources for the surrounding environment [10,11,12], such as solar energy, wind energy, etc., as supplements or substitutes has become increasingly important and necessary. Triboelectric nanogenerators (TENGs) based on coupling contact electrification and electrostatic induction have been proven to be an effective method for harvesting environmental mechanical energy [13,14]. Triboelectric nanogenerators (TENGs) with advantages such as flexible structure, low manufacturing cost, and environmental friendliness provide enormous potential for constructing self-powered sensing systems. However, most of the reported studies currently face the issues of insufficient charge density caused by contact electrification and surface damage caused by friction [14,15].

In order to improve the surface charge density of TENGs, researchers have used physical surface engineering methods, such as pattern arrays (linear, cubic, and pyramid), to improve the efficiency of nanogenerators [16,17]. In addition, surface charge density can also be increased through chemical surface functionalization, which effectively regulates surface potential by changing the exposed functional groups on the surface [18,19]. However, the above two methods also have issues with material wear, tearing after friction, and complex manufacturing processes. In recent years, the emerging charge pump technology has broken through the bottleneck of increasing charge density [20,21]. For example, Xu et al. [22] used charge pumps for the first time in 2018 to increase the amount of charge on metal electrodes and achieve a high charge density up to 1020 µCm^−2^. As a continuous charge source, the charge pump unit generates charges that are extracted from the charge pump and sequentially injected into the conductive layer of the main TENG, decoupling charge density from friction strength. In addition, Bai et al. [23] reported on charge pump-based rotary sliding TENGs, but there is still a problem of surface damage to the friction layer. Lv et al. [24] proposed a soft-contact coplanar charge pumping TENG. The use of a soft-contact central charge pumping TENG enhances durability, injecting charge directly into the main TENG and improving the power generation performance. However, there is still a certain degree of wear in the soft contact of sliding friction; triboelectric nano generators with high durability and high output are urgently needed. 

Herein, a novel coaxial rolling charge pump TENG (CR-TENG) based on wind energy is proposed to enhance the output performance and durability. The rolling friction charge pump TENG directly injects positive and negative charges into the main TENG, which is more wear-resistant compared to sliding friction and greatly increases the charge density and output power. In addition, the charge pumping unit and the main TENG adopt the coaxial design, reducing the complexity of the structural design. On comparing the output performance of the CR-TENG under the initial state, rectifier bridge supplemental charge strategy, and charge pump supplemental charge strategy, it can be concluded that the output performance of the CR-TENG can be improved by 5800% under the charge pump supplemental charge strategy. Moreover, the output performance of the CR-TENG remains stable after 72,000 cycles. The output power of the CR-TENG is 1.21 mW under a load resistance of 3 × 10^7^ Ω. And the CR-TENG can charge a 0.1 μF capacitor to 5 V in just 1.6 s. This work provides new insights for the rotary durable high output charge pump compensating triboelectric nanogenerator and demonstrates the important potential of harvesting environmental energy to supply intelligent IoT nodes.

## 2. Results and Discussion

### 2.1. System Solution

**Figure 1:**
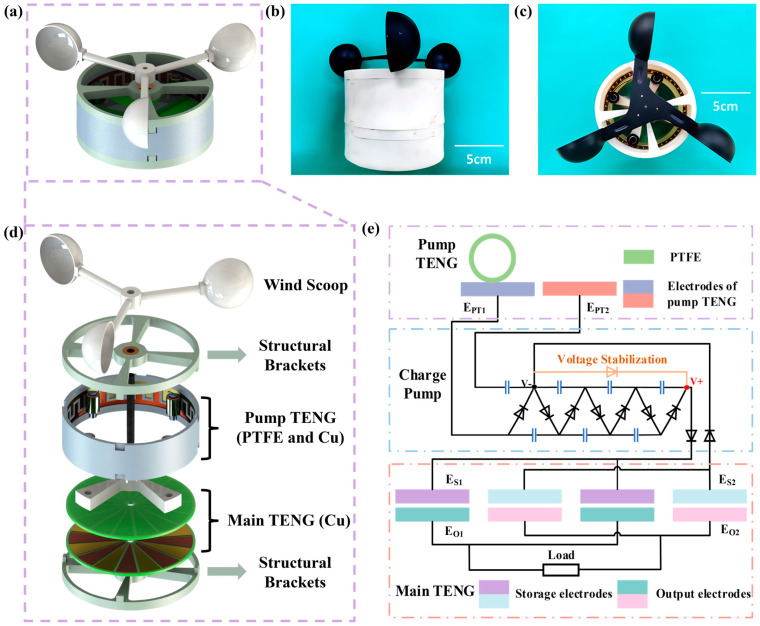
The structural design and working principle of the CR-TENG. (**a**) The schematic diagram of the CR-TENG structure. (**b**,**c**) The physical structure of the CR-TENG. (**d**) Decomposition of the CR-TENG structure. (**e**) Simplified schematic of the circuit of the CR-TENG.

The whole system is divided into two parts, the pump TENG and the main TENG, respectively. The structure of the CR-TENG is schematically shown in Figure 1a, and the physical structure of the CR-TENG is shown in Figure 1b,c. It is mechanically rotated by the environmental wind energy. Moreover, the pump TENG and the main TENG rotate in a coaxial synchronized manner. To reduce frictional resistance and material wear during rotation, the pump TENG adopts a rolling working mode instead of the traditional sliding working mode, and the main TENG adopts a non-contact rotating working mode. To enhance the output performance of the main TENG, the energy generated by the pump TENG is charged directly to the storage electrode of the main TENG after passing through the charge pump circuit. The main TENG generates a lot of inductive charge between the storage electrode and the output electrode during the rotating operation. With the addition of the charge pump, the output performance of the main TENG is far better than the original main TENG and pump TENG.

### 2.2. Structural Design

The pump TENG structure is schematically shown in Figure 1d, with polytetrafluoroethylene (PTFE) attached to the bearing surface. PTFE acts as the negative friction material, and copper acts as the friction material and electrode. A sponge is added between the bearing and PTFE to make better contact between the PTFE and the copper layer. The copper layers are arranged in the way of interdigitated electrodes. And the four negative friction layers are in contact with the copper layer at the same time to superimpose the generated energy to make the pump TENG have a better output.

The structure of the main TENG is shown in the Figure 1d, which consists of just two PCB layers. Wherein, the upper PCB layer is the storage electrode and the lower PCB layer is the output electrode. Each PCB layer is divided into 16 equal-sized sectors, and each is charged by a charge pump, where two neighboring sectors have opposite polarity of charge. During the operation of the main TENG, electrostatic induction occurs between the output electrode and the storage electrode, resulting in the output of powerful energy. It is worth mentioning that the two PCB layers are non-contact with each other, which does not generate frictional resistance and material wear during operation.

When the CR-TENG is in operation, the wind scoop provides the rotational mechanical energy to carry the pump TENG and the main TENG in coaxial rotation. The coaxial design provides more stability and ease of operation than the traditional charge pump replenishment strategy of separating the pump TENG from the main TENG.

### 2.3. Working Principle

**Figure 2:**
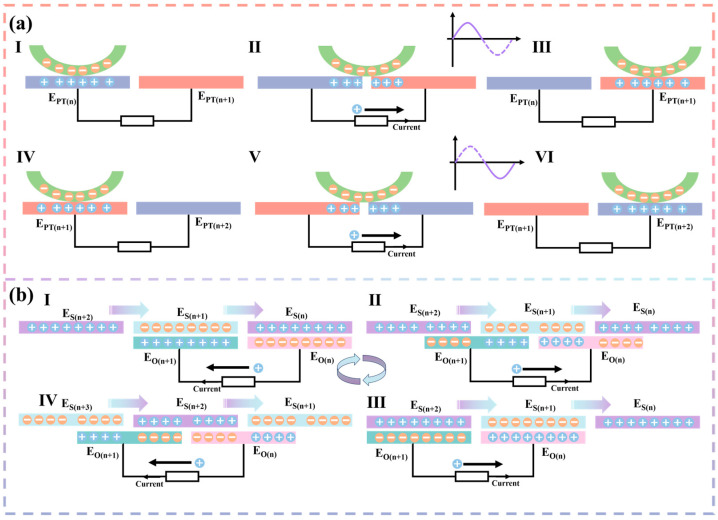
Working principle of the pump TENG and the main TENG. (**a**) Detailed analysis of the working principle of the pump TENG. (**b**) Detailed analysis of the working principle of the main TENG.

The working principle of the whole system is schematically shown in Figure 1e. During the operation of the pump TENG, PTFE rubs against the copper layer in a rolling fashion because PTFE is attached to the bearing. Replacing sliding friction with rolling friction greatly reduces the frictional resistance during operation and does not change the contact area, which greatly increases the output performance of the pump TENG. A detailed schematic of the operating principle of the pump TENG is shown in Figure 2a. The I state is the initial state, where PTFE contacts E_PT(n)_ to reach an electrostatic equilibrium. As PTFE rolls, PTFE is between the two electrodes as shown in state II. Due to electrostatic equilibrium, positive charges are transferred from E_PT(n)_ to EP_T(n+1)_, resulting in a current. State III is that PTFE is completely in contact with E_PT(n+1)_, and the positive charge realizes complete transfer and reaches the electrostatic equilibrium state. It is assumed that the current generated by the positive charge transfer when PTFE moves from E_PT(n)_ to E_PT(n+1)_ is the forward current. From state IV to state VI, the contact state of PTFE goes from E_PT(n+1)_ to E_PT(n+2)_ and the positive charge transfer goes from E_PT(n+1)_ to E_PT(n+2)_. Therefore, the current generated from state IV to state VI is a negative current. State I to state VI is one cycle, so state VI is equivalent to state I. The energy generated by the pump TENG is recharged through a standard charge pump to the storage electrode of the main TENG.

A detailed schematic on the working principle of the main TENG is shown in Figure 2b. The storage electrodes have a much higher charge density due to the charge input from the pump TENG. Suppose E_S(n)_ is supplemented with a positive charge and E_S(n+1)_ is supplemented with a negative charge. From state I to state IV is one work cycle. State I serves as the initial state, where the storage electrode reaches electrostatic equilibrium with the output electrode. As the storage electrode rotates, the output electrode is located between the storage electrodes as shown in state II. Due to the electrostatic effect, positive charges will flow from E_O(n+1)_ into E_O(n)_, resulting in a current. In state III, the positive charge will flow completely into E_O(n)_. Assume that the current generated when a positive charge flows from E_O(n+1)_ to E_O(n)_ is a forward current. Then, in state IV, positive charges flow from E_O(n)_ into E_O(n+1)_ as the storage electrode rotates. In state I, the positive charge is completely transferred into E_O(n+1)_. So, from state III to state I, the current generated is negative current.

### 2.4. Experimentation and Analysis

**Figure 3:**
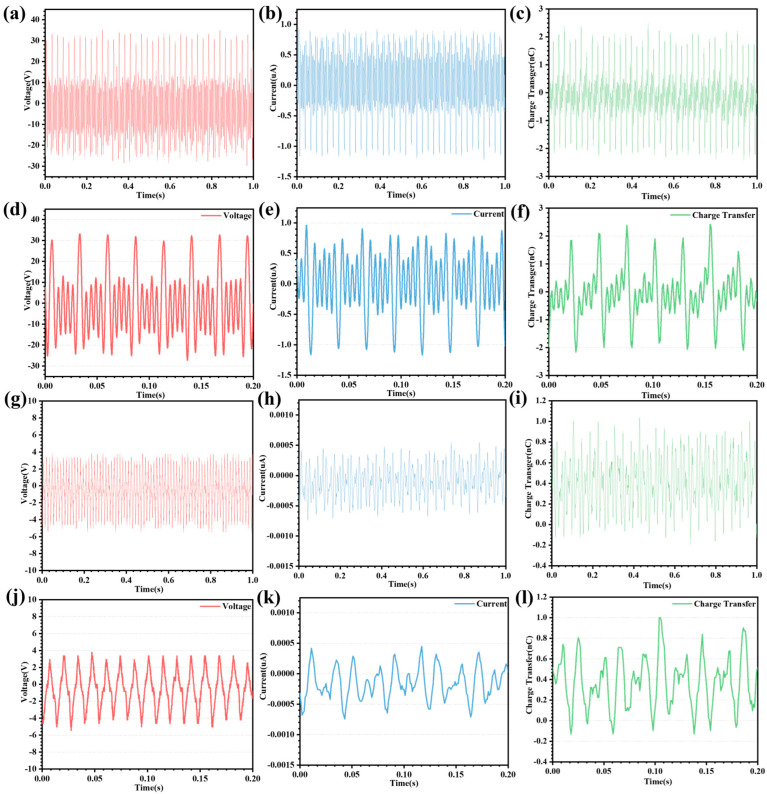
Output performance of the pump TENG and main TENG in the initial state. (**a**–**c**) The pump TENG output voltage, current, and charge transfer curves in the initial state. (**d**–**f**) Detailed curves of the output voltage, current, and charge transfer sections of the pump TENG in the initial state. (**g**–**i**) The main TENG output voltage, current, and charge transfer curves in the initial state. (**j**–**l**) Detailed curves of the output voltage, current, and charge transfer sections of the main TENG in the initial state.

To investigate the output performance of the pump TENG and the main TENG before the supplemental charge strategy, separate tests are performed under the same conditions. The CR-TENG is tested at 540 rpm. The test results of the pump TENG voltage, current, and charge transfer are shown in Figure 3a–c. To better investigate the output performance of the pump TENG in terms of voltage, current, and charge transfer, some of the output curves are shown in Figure 3d–f. As can be seen from the output results, one higher sine wave and four lower sine waves form a cycle. This is because one set of interdigitated electrodes consists of five sets of copper electrodes, and a total of four sets of interdigitated electrodes are on the inner wall of the housing. Four sets of interdigitated electrodes are placed at equal intervals to ensure the consistency of the pump TENG output. The interdigitated electrode is non-contact with the PTFE when the PTFE is rolled over the blank area. In this case, the charges in the interdigitated electrodes equalize at a very fast rate, generating a large amount of charge transfer, which in turn generates a current. Similarly, when the PTFE rolls to the point where it is about to contact the interdigitated electrode, a large amount of charge transfer also occurs. Therefore, the output voltage peak-to-peak, current peak-to-peak, and charge transfer of pump TENG at 540 rpm are 64.94 V, 2.21 μA, and 4.58 nC. However, the effective contact area is only 2 cm^2^, resulting in a pump TENG output performance that is far from adequate. The output performance of the main TENG is even worse in the absence of a replenishment strategy. The main TENG output performance curves are shown in Figure 3g–i, and the partial output performance curves are shown in Figure 3j–l. The output voltage peak-to-peak, current peak-to-peak, and charge transfer of the main TENG are 10.05 V, 0.01 μA, and 1.14 nC, respectively, at a rotational speed of 560 rpm. The reason the main TENG output performance is so poor is that the two PCB layers have only copper electrodes and no friction material. The absence of charge replenishment in the upper PCB layer results in a low surface charge density, which generates less induced charge with the lower PCB layer. As a result, the output performance of the main TENG is poor until a charge replenishment strategy is performed. To improve the output performance of the main TENG, it is necessary to add charge into the storage electrode to increase the charge density on the surface of the storage electrodes.

**Figure 4:**
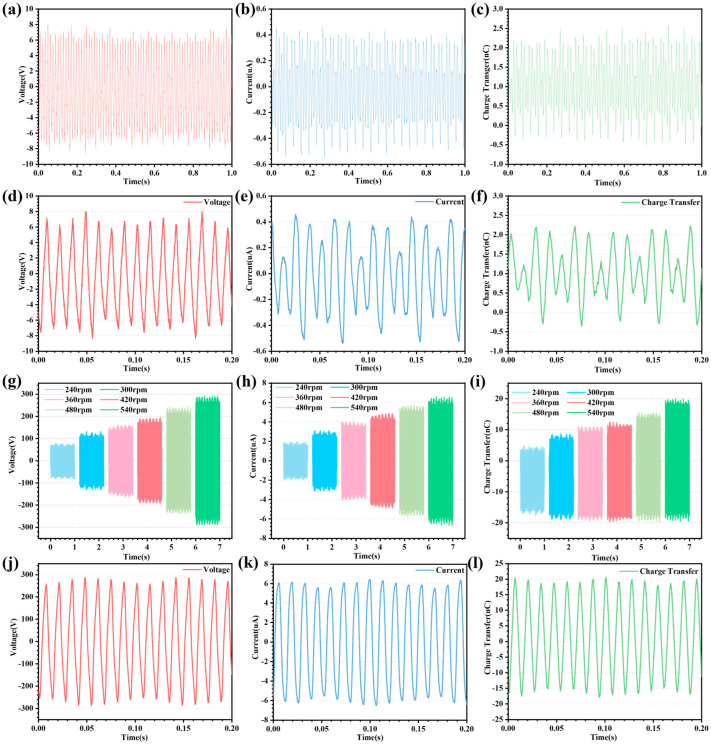
Output performance of the CR-TENG after the supplemental charge strategy. (**a**–**c**) Curves of output voltage, current, and charge transfer of the CR-TENG after the rectifier bridge supplemental charge strategy. (**d**–**f**) Some detailed curves of the output voltage, current, and charge transfer of the CR-TENG after the rectifier bridge supplemental charge strategy. (**g**–**i**) Comparison of output voltage, current, and charge transfer of the CR-TENG at different speeds (240, 300, 360, 420, 480 and 540 rpm) after the charge pump supplemental charge strategy. (**j**–**l**) Detailed curves of output voltage, current, and charge portion of the CR-TENG at 540 rpm speed after the charge pump supplemental charge strategy.

The simplest supplemental charge strategy for the main TENG is rectifier bridge supplementation. The output curves of the main TENG at 540 rpm and the rectifier bridge supplemental charge strategy are shown in Figure 4a–c, and some of the curves are shown in Figure 4d–f. The output voltage peak-to-peak, current peak-to-peak, and charge transfer of the main TENG after the rectifier bridge replenishment charge strategy are 17.17 V, 1.13 μA, and 2.71 nC, respectively. Compared to the previous output of the main TENG without the rectifier bridge replenishment strategy, the performance improvement is about 70%. As can be seen in Figure 4a–f, the output of the main TENG is unstable after the rectifier bridge replenishment strategy. This occurs because the rectifier bridge supplemental charge strategy charge input is unstable, resulting in the storage electrode charge density also being unstable. Another reason is that the rectifier bridge supplemental charge strategy simply charges the positive or negative charges, resulting in half of the storage electrodes being inactive. Therefore, using the rectifier bridge supplemental charging strategy to enhance the output performance of the main TENG is not a good choice.

To investigate the effect of the charge pump supplemental charging strategy on the output performance of the main TENG, the output results of the main TENG at different rotational speeds and charge pump supplemental charging strategies are shown in Figure 4g–i, and some detailed curves are shown in Figure 4j–l. From the output results seen in Figure 4g–i, the output performance of the main TENG tends to increase linearly with the increase in rotational speed. The output voltage peak-to-peak, current peak-to-peak, and charge transfer of the main TENG at 540 rpm and with the charge pump supplemental charge strategy are 582.00 V, 13.53 μA, and 39.18 nC, respectively. The main TENG improves output performance by roughly 5800% in comparison to the strategy without charge pump supplemental charge. The reason for such a large increase in output performance is that the charge pump replenishment strategy allows for a stable charge input, resulting in a highly stable output from the main TENG. Another reason is that neighboring storage electrodes are each charged with a different polarity, causing a substantial charge transfer between the output electrodes. As a result, the output performance of the main TENG is greatly increased after the charge pump replenishment strategy. It is worth mentioning that the pump TENG and the main TENG rotation method adopts coaxial rotation, which greatly reduces the complexity of the structure.

**Figure 5:**
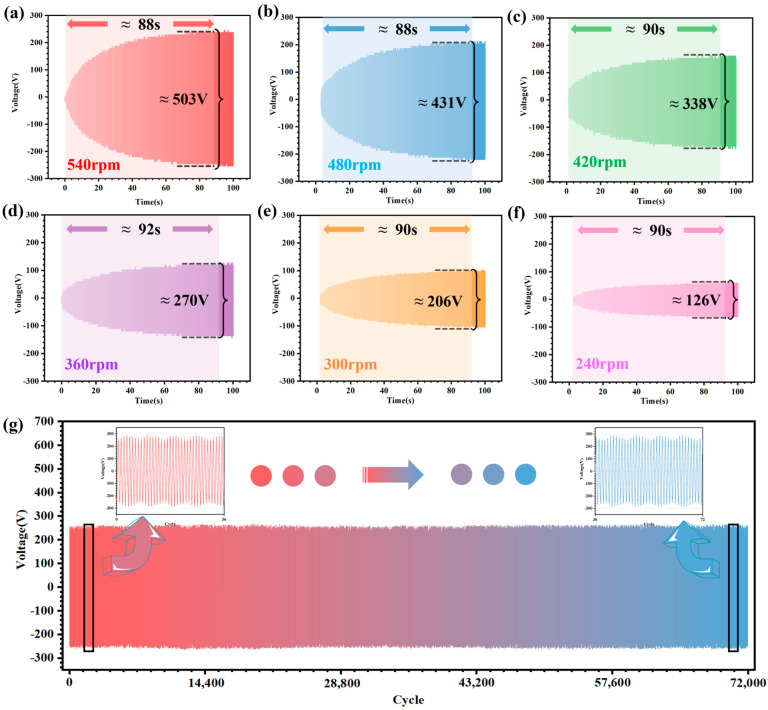
Stability of the CR-TENG after the charge pump supplemental charge strategy. (**a**–**f**) Output voltage curves of the CR-TENG from initial state to saturation state at different rotational speeds (240, 300, 360, 420, 480, and 540 rpm). (**g**) Curve output by the CR-TENG operating at 540 rpm for 72,000 cycles (where the top left graph shows the output voltage curve of the CR-TENG in the initial state, and the top right graph shows the output voltage curve after 72,000 cycles of operation).

To study the relationship between the time required for the storage electrode charge density to reach saturation and the CR-TENG rotational speeds, the output performance of the main TENG is tested at different rotational speeds. Figure 5a–f shows the output voltage profile of the main TENG at different speeds (the CR-TENG rotational speeds are 240, 300, 360, 420, 480, and 540 rpm). From the output results, it is known that the time for the storage electrode charge density to saturate is approximately 90 s, regardless of the rotational speed. The charge density of the storage electrodes reaching saturation is not the same at different rotational speeds, resulting in different main TENG output performances. This is due to the fact that the pump TENG decreases with rotational speed, resulting in a decrease in output performance. This in turn leads to a reduction in the input charges to the charge pump supplemental charge strategy. And this ultimately leads to a decrease in the saturation charge density of the storage electrodes.

For the stability study of the CR-TENG output performance, a long-time stability test is done at a speed of 540 rpm. The output result curve is shown in Figure 5g. It can be concluded that the output performance of the main TENG is almost the same as that of the initial state after 72,000 cycles. The experimental results fully confirm that the CR-TENG has excellent output stability and can perfectly cope with long working hours.

**Figure 6:**
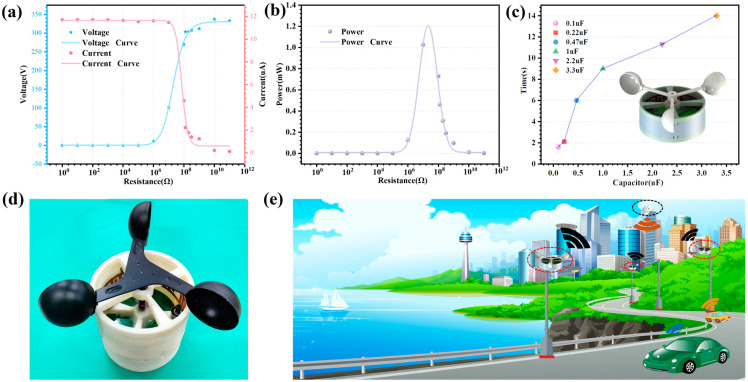
Applications of the CR-TENG. (**a**) Output voltage and current of the CR-TENG with different load resistors connected. (**b**) The CR-TENG output power with different load resistors connected. (**c**) Charging time of different capacitors by the CR-TENG at a speed of 540 rpm. (**d**) The physical diagram of the CR-TENG. (**e**) The expected application of the CR-TENG in the field of self-powered monitoring and wireless transmission of information in urban environments(Red circle is CR-TENG, and black circle is wireless receiving device).

In order to study the output performance of the CR-TENG more deeply, the CR-TENG is connected to load resistors of different resistance values to test its output performance. By connecting the CR-TENG to different load resistors, the output voltage and current results are shown in Figure 6a. When the load resistor resistance is 10^0^–10^5^ Ω, the CR-TENG output voltage is extremely low. However, the CR-TENG output voltage starts to increase when the load resistor resistance is increased to 10^6^ Ω. And when the resistance value is increased to 10^10^ Ω, the output voltage grows to 337 V, after which it equalizes. Regarding the output current of the CR-TENG, the output current is 11.71 μA when the load resistance is 10^0^–10^7^ Ω. However, the output current drops sharply when the load resistance value is 10^7^–10^9^ Ω. After that, it tends to equalize. Based on the peak output power formula:

(1) $$P_{peak}=\frac{U_{peak}{}^{2}}{R}$$

where $U_{peak}$ is the peak-to-peak CR-TENG output voltage and R is the load resistor resistance. The output result is shown in Figure 6b; when the load resistance is 10^0^–10^5^ Ω, the CR-TENG output power is extremely low. However, the output power rises sharply when the load resistance is 10^5^–10^7^ Ω. The output power is 1.03 mW when the load resistance is 10^7^ Ω. Based on the curve fitting, it can be concluded that the output power is maximum when the load resistance is 3 × 10^7^ Ω, which is 1.21 mW. By using the CR-TENG to charge capacitors of different capacities, it is used to verify the output performance of the CR-TENG. The result of the charging time of capacitor by the CR-TENG at a speed of 540 rpm is shown in Figure 6c. The results demonstrate that the CR-TENG takes just 1.6 s to charge a 0.1 μF capacitor to 5 V and 14 s for a 3.3 μF capacitor. Figure 6d shows the physical diagram of the CR-TENG. Figure 6e shows the expected application of the CR-TENG in the field of self-powered monitoring and wireless transmission of information in urban environments. The energy harvested by the CR-TENG can power wireless transmission modules and a variety of sensors, such as temperature, humidity, or wind speed sensors. At the same time, the data information collected by the sensors is transmitted wirelessly, ultimately achieving self-powered wireless monitoring of the urban environment.

## 3. Conclusions

We have designed a novel coaxial rolling charge pump TENG (CR-TENG) based on wind energy to enhance the output performance and durability. The CR-TENG consists of the pump TENG and main TENG. The pump TENG working mode adopts rolling rotation friction, which greatly reduces friction resistance and friction material wear. The main TENG operates in a non-contact rotation mode, which also has an excellent positive effect on the wear of friction material and the reduction of friction resistance. The coaxial rotation of the pump TENG and the main TENG greatly reduces the complexity of the CR-TENG structure, making the structure easier to install and more stable. The circuitry of the CR-TENG works in such a way that the pump TENG is supplemented with positive and negative charges by a charge pump into the storage electrodes of the main TENG. The storage electrodes in turn induce a charge with the output electrodes (charge transfer occurs). On comparing the output performance of CR-TENG under the initial state, rectifier bridge supplemental charge strategy, and charge pump supplemental charge strategy, it can be concluded that the output performance of the CR-TENG can be improved by 5800% under the charge pump supplemental charge strategy. Moreover, the output performance of the CR-TENG remains stable after 72,000 cycles. The CR-TENG can produce a power of 1.21 mW with a load resistance of 3 × 10^7^ Ω. And the CR-TENG can charge a 0.1 μF capacitor to 5 V in just 1.6 s. The CR-TENG is not only a high-performance device in the field of energy harvesting, but also has promising applications in the fields of self-powered monitoring of urban environments and wireless transmission.

## 4. Experimental Section

### 4.1. Fabrication of Interdigitated Electrodes

The contours of the interdigitated electrodes are engraved on the copper film using a laser engraver (D1 Pro XTOOL). Then use a knife or scissors to cut the process. After making 4 sets of interdigitated electrodes, the electrodes are connected in series and pasted on the inner wall of the TENG housing. There are 4 groups of interdigitated electrodes in the CR-TENG. Each group of interdigitated electrodes contains 5 pairs of interdigitated electrodes. The spacing between the electrodes in each pair of interdigitated electrodes is 2 mm. And each interdigitated electrode has a dimension of 15 mm × 5 mm.

### 4.2. Fabrication of Storage and Output Electrodes

In fact, both the storage and output electrodes are PCBs with copper areas. The reason for the different names is because they have different functions in the CR-TENG.

### 4.3. Statistical Analysis

Statistics are performed using the software Origin 2018 (Origin Lab Corporation, Northampton, MA, USA).

## Data Availability

The data presented in this study are available on request from the corresponding author.
